# Unusual *Talaromyces marneffei* and *Pneumocystis jirovecii* coinfection in a child with a *STAT1* mutation: A case report and literature review

**DOI:** 10.3389/fimmu.2023.1103184

**Published:** 2023-02-20

**Authors:** Qin Yang, Chendi Yu, Yue Wu, Ke Cao, Xiaonan Li, Weiguo Cao, Lichao Cao, Shenrui Zhang, Ying Ba, Yuejie Zheng, Hezi Zhang, Wenjian Wang

**Affiliations:** ^1^ Department of Respiratory Diseases, Shenzhen Children’s Hospital Affiliated to Shantou University Medical College, Shenzhen, China; ^2^ Department of Research and Development, Shenzhen Nuclear Gene Technology Co., Ltd., Shenzhen, China; ^3^ Department of Pharmacy, Shenzhen Children’s Hospital Affiliated to Shantou University Medical College, Shenzhen, China; ^4^ Clinical Laboratory, Shenzhen Children’s Hospital Affiliated to Shantou University Medical College, Shenzhen, China; ^5^ Department of Radiology, Shenzhen Children’s Hospital Affiliated to Shantou University Medical College, Shenzhen, China

**Keywords:** *Talaromyces marneffei*, *Pneumocystis jirovecii*, coinfection, *STAT1*, metagenome next-generation sequencing

## Abstract

*Talaromyces marneffei* and *Pneumocystis jirovecii* are the common opportunistic pathogens in immunodeficient patients. There have been no reports of *T. marneffei* and *P. jirovecii* coinfection in immunodeficient children. Signal transducer and activator of transcription 1 (*STAT1*) is a key transcription factor in immune responses. *STAT1* mutations are predominately associated with chronic mucocutaneous candidiasis and invasive mycosis. We report a 1-year-2-month-old boy diagnosed with severe laryngitis and pneumonia caused by *T. marneffei* and *P. jirovecii* coinfection, which was confirmed by smear, culture, polymerase chain reaction and metagenome next-generation sequencing of bronchoalveolar lavage fluid. He has a known *STAT1* mutation at amino acid 274 in the coiled-coil domain of *STAT1* according to whole exome sequencing. Based on the pathogen results, itraconazole and trimethoprim-sulfamethoxazole were administered. This patient’s condition improved, and he was discharged after two weeks of targeted therapy. In the one-year follow-up, the boy remained symptom-free without recurrence.

## Introduction

*Talaromyces marneffei* is one of the common opportunistic pathogens prevalent in southeast Asia (1). *Pneumocystis jirovecii* most commonly affects immunocompromised individuals worldwide (2). Signal transducer and activator of transcription 1 (*STAT1*) is the primary transcription factor downstream of interferons and cytokines, so it plays a major role in normal immune responses, particularly to viral, bacterial, and fungal pathogens (3). *STAT1* mutations have been identified worldwide since their discovery in 2003. The clinical manifestations associated with *STAT1* mutations are unexpectedly broad, including chronic mucocutaneous candidiasis, and susceptibility to various viruses, bacteria, and invasive fungi (4). *T. marneffei* and *P. jirovecii* infection have been reported separately in individuals carrying *STAT1* mutations (5, 6). Here, we present a boy carrying a known *STAT1* mutation, with complicated and repeated infections characterized by rare *T. marneffei* and *P. jirovecii* coinfection. To the best of our knowledge, this is the first case of such mixed infection in immunodeficient children.

## Case presentation

**Figure 1:**
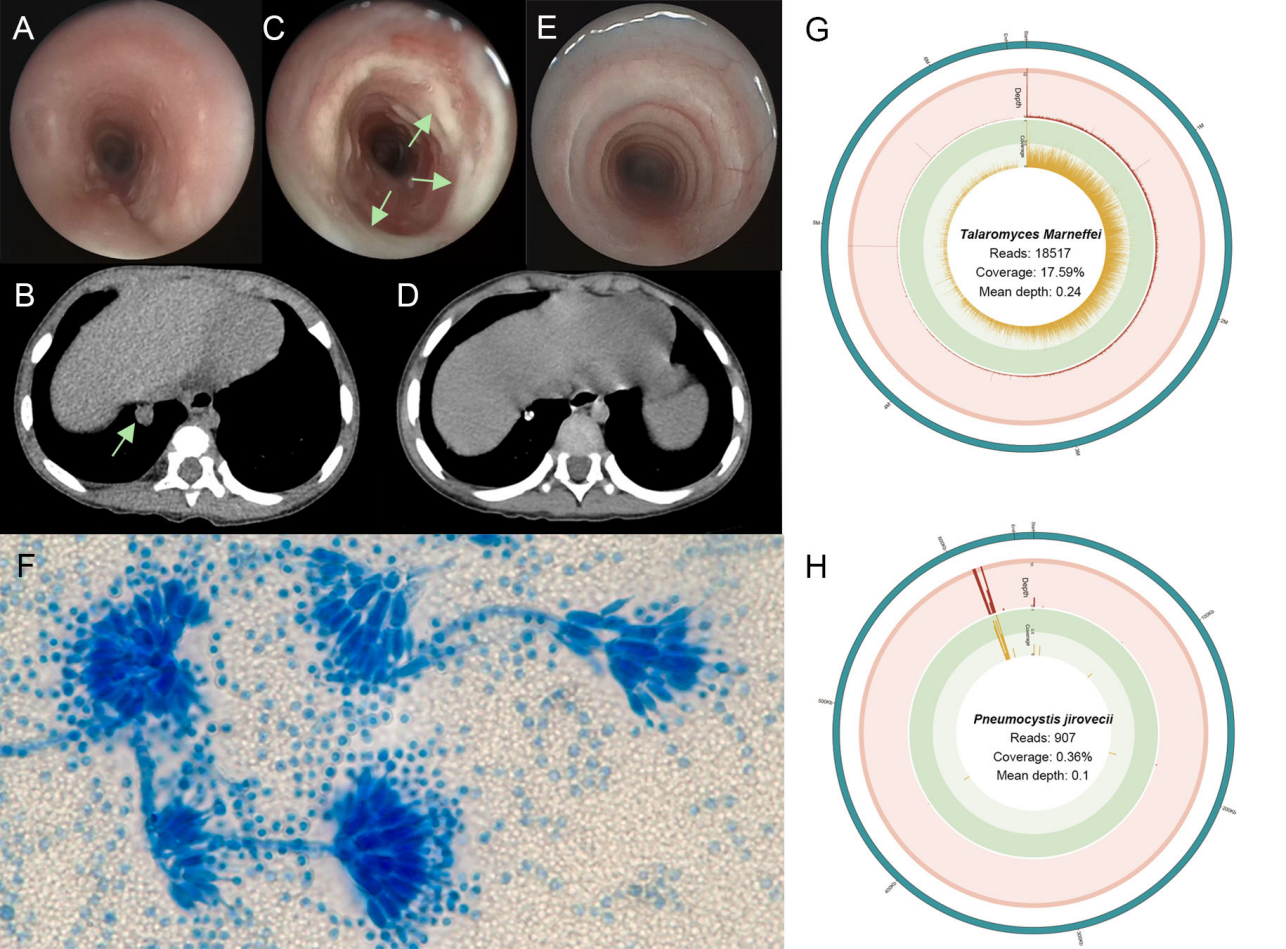
**(A)** The first bronchoscope showing a little mucus in the airway. **(B)** Three-dimensional computed tomography reconstruction of lung window showing a round and high-density shadow in the basal segment (arrows). **(C)** The bronchoscopic image showing plenty of white secretion in the tracheal inner membrane (arrows). **(D)** After one year, chest computed tomography showed the nodule shadows was smaller than before, and the calcification was obvious. **(E)** One year after treatment, tracheoscopy showed no secretion adhesion in the trachea. **(F)** The lactophenol cotton blue of lavage fluid-stained slide on day 33 showing *Talaromyces marneffei* with broom-like branches (oil immersion lens, 1000× magnification). **(G)** *T. marneffei* coverage and depth in BALF metagenome next-generation sequencing (mNGS). **(H)** *Pneumocystis jirovecii* coverage and depth in BALF mNGS.

**Figure 2:**
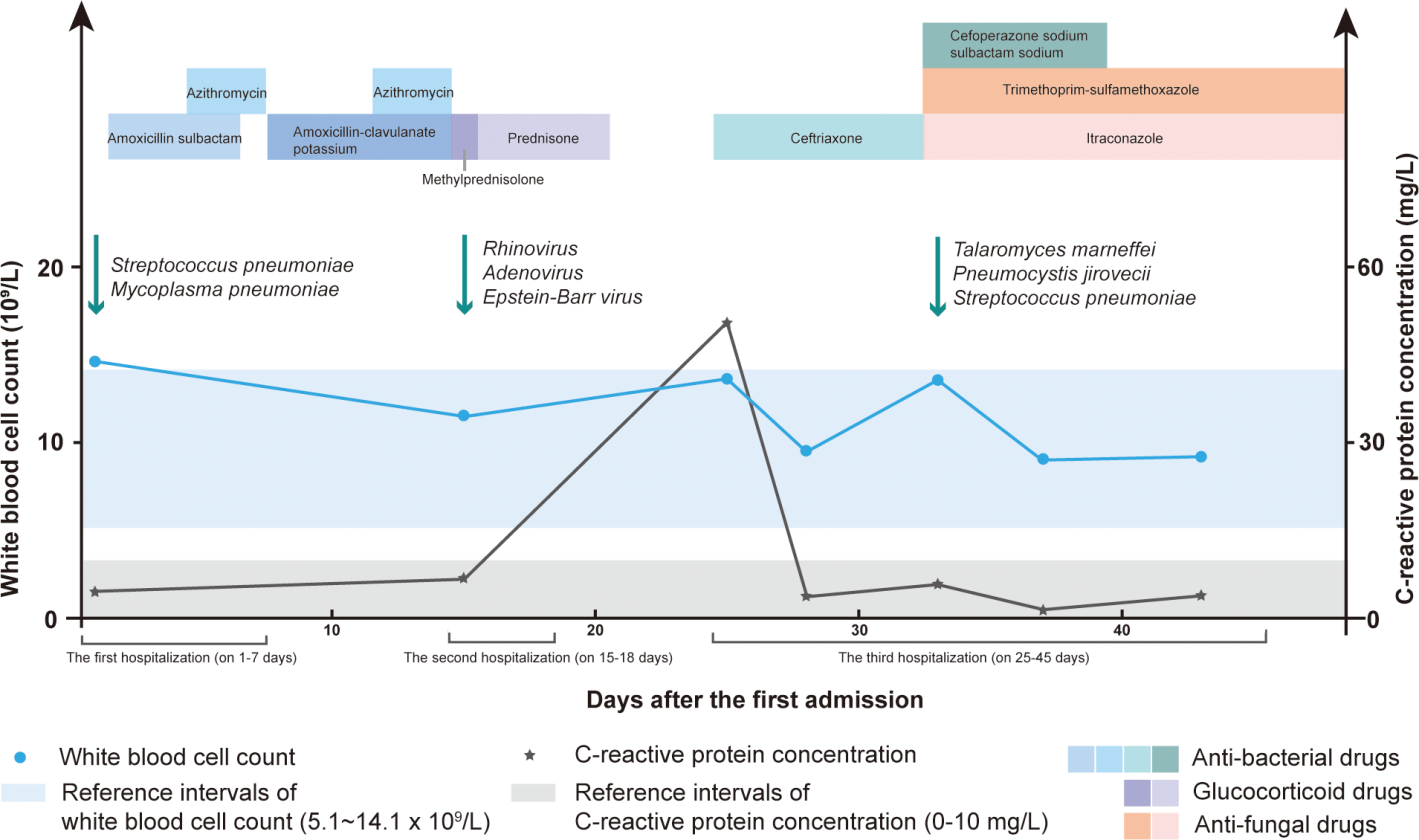
The white blood cell (WBC) count, C-reactive protein (CRP) concentration, detected pathogens and therapy methods during the hospital stay.

A 1-year-2-month-old boy was admitted to our hospital because of a cough and wheezing for half a month. On admission, the child had dyspnea, wheezing, and moist rales can be heard in the lungs. Laboratory data revealed the white blood cell (WBC) count of 17.89×10^9^/L and the C-reactive protein (CRP) concentration of 8.65 mg/L. Electronic bronchoscope showed endobronchial inflammation (**Figure 1A**). Electronic fiber laryngoscope indicated laryngitis. Chest computed tomography (CT) revealed inflammatory lesions, nodules, and swelling lymph nodes. The bronchoalveolar lavage fluid (BALF) polymerase chain reaction (PCR) test of *Mycoplasma pneumoniae* was weakly positive. The BALF culture showed *Streptococcus pneumoniae* (amoxicillin sensitive). After admission, the patient was given amoxicillin sulbactam (on days 2-6) and azithromycin (on days 5-7) for anti-infective therapy (**Figure 2**). He was discharged on day 8 with amoxicillin-clavulanate potassium (on days 8-14) and azithromycin (on days 12-14). He returned on day 15 for cough, wheezing, and trachyphonia, with a temperature of 37.0°C. The throat swab PCR tests showed positive *Rhinovirus* (RHV), *Adenovirus*, and *Epstein-Barr virus* (EBV). He was diagnosed with acute laryngitis. Anti-infective therapy was switched to methylprednisolone (on day 15), followed by prednisone (on days 16-20) (**Figure 2**). He was discharged home on day 18 with intermittent coughing.

One week later, he returned because of shortness of breath, aggravated trachyphonia, and fever. Upon admission, CRP concentration was elevated (50.84mg/L) (**Figure 2**). On day 26, Chest CT showed multiple enlarged necrotic lymph nodes in the hilus and mediastinum and a high-density round shadow in the basal segment in the right lung inferior lobe (**Figure 1B**). He was given ceftriaxone (on days 25-32) as an antibacterial treatment. But the symptoms did not improve. On day 32, the second bronchoscope observed plenty of mucus in the inner tracheal membrane (**Figure 1C**). Various test methods were executed immediately to identify the pathogens. The BALF smear and culture revealed *T. marneffei* (**Figure 1F**). By the same BALF token, PCR tests for targeted pathogen detection and metagenome next-generation sequencing (mNGS) for unbiased pathogen detection were performed. The PCR results revealed *P. jirovecii*. BALF mNGS identified 1515121 microbial sequence reads, of which 18517 reads and 907 reads mapped to *T. marneffei* (**Figure 1G**) and *P. jirovecii* (**Figure 1H**), respectively. 158 reads aligned to *S. pneumoniae.* Following the pathogen results, cefoperazone sodium sulbactam sodium (on days 33-39), itraconazole (on days 33-45), and trimethoprim-sulfamethoxazole (on days 33-45) were commenced as the targeted antimicrobial therapy (**Figure 2**).

**Figure 3:**
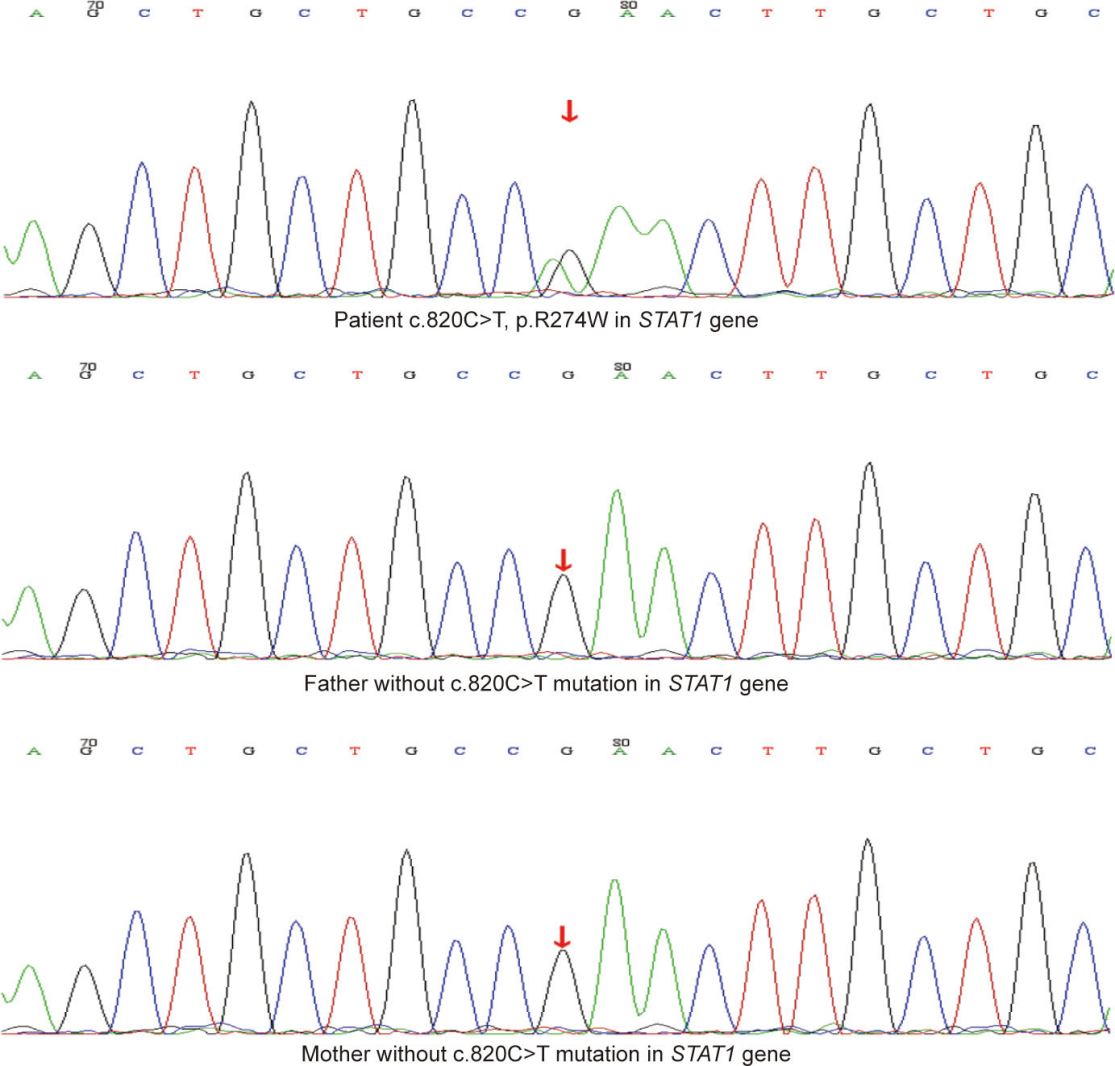
The Sanger sequencing results of the mutation site (c.820C>T, p.R274W) in *STAT1* gene of the patient and his family.

He had no history of exposure to wild bamboo rats, and his HIV test result was negative. His humoral immunity of IgG, IgA, IgM, IgE, C3, and C4 was normal. The fine immunoassay of lymphocytes showed impaired B cell differentiation, and the number of CD4 T cells and natural killer (NK) cells were 2365.88 and 63.80 cells/ul, respectively (**Supplementary Table 1**). Considering that *T. marneffei* and *P. jirovecii* are the main opportunistic pathogens in patients with immune deficiency, genetic test was recommended to clarify the genetic risk of immunodeficiency. Whole-exome sequencing (WES) results identified a missense variant c.820C>T (p.R274W) in the *STAT1* gene. According to the American College of Medical Genetics and Genomics standard, this mutation should be categorized as pathogenic, with proofs of PS4+PM1+PM2+PM5+PM6+PP3. Verification of this variant site using sanger sequencing showed negative results in his family, and it was a *de novo* variant in this patient (**Figure 3**). *STAT1* mutation can inhibit the differentiation of T cells into T-helper 17 (Th17) cells, resulting in a decrease in IL-17 secretion, which is closely related to chronic mucocutaneous candidiasis and invasive mycosis. This boy’s evident decline in Th17 cells through flow cytometry confirmed the consistency between gene mutation and phenotype (**Supplementary Table 1**).

On day 45, his symptoms improved significantly. The patient was discharged with itraconazole and trimethoprim-sulfamethoxazole until now. One year after discharge, the chest CT image was improved, indicating calcification of the primary lesion (**Figure 1D**). The bronchoscope showed that the white mucus in the tracheal membrane disappeared totally (**Figure 1E**).

## Discussion

To the best of our knowledge, this is the first coinfection case with *T.marneffei* and *P. jirovecii* in immunodeficient children. The mixed infection cases related to *T.marneffei* or *P. jirovecii* in HIV-negative children are listed in **Table 1**. 11 cases (68.8%) and 9 cases (56.3%) with *T.marneffei* infection (16 cases) showed bacteria and virus mixed infection, respectively. The reported bacteria mainly contained *S. pneumoniae* (n=3, 18.8%), *Klebsiella pneumoniae* (n=2, 12.5%), *Moraxella catarrhalis* (n=2, 12.5%), *Mycobacterium Tuberculosis* (n=2, 12.5%), and *M. pneumoniae* (n=2, 12.5%). *Cytomegalovirus* (CMV, n=2, 12.5%), EBV (n=3, 18.8%), *Hepatitis B virus* (n=2, 12.5%), and RHV (n=2, 12.5%) were more common in the mixed virus infection. 6 cases (40%) with fungi coinfection of *T.marneffei* all belong to *Candida* spp. More than half of *P. jirovecii* mixed infection cases (n=16) showed coinfection with bacteria (62.5%, n=10) or virus (62.5%, n=10). The most frequent bacterium was *Haemophilus influenzae* (n=3, 18.8%), followed by *Pseudomonas aeruginosa* (n=2, 12.5%) and *S. pneumoniae* (n=2, 12.5%). The virus included in *P. jirovecii* cases were CMV (n=4, 25%) and RHV (n=4, 25%). Only two children (13.3%) showed mixed fungi infection, caused by *Aspergillus fumigatus*. In this case, testing results of BALF identified bacterial infection of *S. pneumoniae* and fungi infection of *T.marneffei* and *P. jirovecii*. The rare coinfection of *T.marneffei* and *P. jirovecii* provided a reference for higher awareness of mixed fungi infections.

**Table 1:** Reported coinfection cases of *T. marneffei* or *P. jirovecii* in children.

No.	Sex	Age	Mixed infections	Methods	Clinical features	Genetic mutation	Antimicrobial treatments	Outcome	Ref.
P1	M	1y2m	*T. marneffei, P. jirovecii*, *S. pneumoniae*	Smear Culture PCR mNGS	Fever, cough, shortness of breath, trachyphonia, laryngitis, pneumonia	*STAT1* mutation	ITZ, TMP-SMX, Ceftriaxone, Cefoperazone sodium sulbactam sodium	Improved	This study
P2	F	7y11m	*T. marneffei, S. pneumoniae, H. influenzae, Moraxella catarrhalis*, EBV	Smear Culture mNGS	Fever, pneumonia, weight loss, skin lesions, CMC, hepatosplenomegaly, lymphadenopathy	*STAT1* mutation	VCZ, ITZ, AmB, Isoniazid, Fluconazole, Rifampicin, Pyrazinamide, Linezolid	Improved	(7)
P3	M	8y6m	*T. marneffei, Candida albicans, M. catarrhalis, H. influenzae, Staphylococcus aureus*, RHV	Smear Culture mNGS	Fever, pneumonia, weight loss, CMC, osteolytic lesions, lymphadenopathy, hepatosplenomegaly, lymphopenia	*STAT1* mutation	VCZ, ITZ	Improved	(7)
P4	F	2y4m	*T. marneffei, M. pneumoniae*, EBV	Smear Culture PCR	Fever, weight loss, lower limbs swelling, hemophagocytic syndrome, hepatosplenomegaly	–	VCZ	Improved	(8)
P5	F	9y1m	*T. marneffei, Candida* spp.*, M. tuberculosis*, EBV	–	Lymphadenectasis, chronic lung disease, hepatosplenomegaly, hypothyroidism	*STAT1* mutation	ITZ, AmB, SMX, Oseltamivir	Improved	(9)
P6	M	1y5m	*T. marneffei, K. pneumoniae, Enterobacter cloacae, Burkholderia cepacian*, CMV	Smear Culture mNGS	Fever, pneumonia, weight loss, thrush, diarrhea, hepatomegaly, hepatic failure, ARDS	*ADA* mutation	VCZ, AmB, Isoniazid, Rifampicin	Death	(7)
P7	M	1y1m	*T. marneffei, Salmonella typhimurium*, CMV	Smear Culture mNGS	Fever, pneumonia, weight loss, hypothyroidism, hepatosplenomegaly, lymphadenopathy	*CD40LG* mutation	VCZ, ITZ, AmB	Death	(7)
P8	F	2y5m	*T. marneffei, M. pneumoniae*	Smear Culture mNGS	Fever, pneumonia, weight loss, intracranial infection, respiratory failure, lymphadenopathy	*STAT3* mutation	VCZ, ITZ, AmB, Micafungin, Isoniazid, Rifampicin, Pyrazinamide	Death	(7)
P9	M	8m	*T. marneffei*, RHV	Smear Culture mNGS	Fever, pneumonia, hematuresis, rash, edema, diarrhea, hepatosplenomegaly	*IL2RG* mutation	VCZ, ITZ, AmB	Death	(7)
P10	M	4m	*T. marneffei, Candida parapsilosis, M. Tuberculosis*, RHV	Smear Culture mNGS	Fever, pneumonia, weight loss, MODS, peritonitis hepatosplenomegaly, HLH	*IL2RG* mutation	VCZ, Isoniazid, Rifampicin, Pyrazinamide, Linezolid	Death	(7)
P11	M	4m	*T. marneffei, C. albicans, K. pneumoniae, Escherichia coli, P. aeruginosa*, HBV	Smear Culture PCR	Erythema and papules on whole-body skin	–	VCZ	Death	(8)
P12	F	3.5m	*T. marneffei, C. albicans, Staphylococcus hominis.*, HSV	Smear Culture PCR	Fever, weight loss, hepatosplenomegaly, swelling in lower limbs, hemophagocytic syndrome	–	VCZ	Death	(8)
P13	M	2y4m	*T. marneffei, C. albicans*	Smear Culture PCR	Fever, cough, weight loss, gasp, aerothorax, empyema	–	VCZ	Death	(8)
P14	F	2y	*T. marneffei, S. pneumoniae*	Culture	Fever, cough, abdominal, jaundice	*STAT3* mutation	VCZ, AmB	Death	(10)
P15	M	2y6m	*T. marneffei, C. tropicalis*	Smear Culture PCR	Fever, weight loss, bellyache, lymph node enlargement, hepatosplenomegaly	–	VCZ	–	(8)
P16	M	1y7m	*T. marneffei*, HBV	Smear Culture PCR	Fever, cough, weight loss, lymph node enlargement (neck, armpit, mediastinal), hemophagocytic syndrome	–	VCZ	–	(8)
P17	M	4m	*P. jirovecii, Stenotrophomonas maltophilia*, CMV	mNGS	Fever, cough, pneumonia	*CD40LG* mutation	SMX, Ganciclovir	Improved	(11)
P18	M	10m	*P. jirovecii*, CMV	PCR mNGS	Fever, cough, tachypnea, cyanosis, diffuse nonsegmental ground glass opacity in both lungs, left axillary lymph node calcification	*CD40LG* mutation	TMP-SMX, Meropenem, Ganciclovir	Improved	(12)
P19	M	2m	*P. jirovecii*, CMV	PCR mNGS	Fever, scattered bleeding spots and mild skin yellowing, acute laryngitis, hydrocele, cholestatic hepatitis, ITP	–	Dexamethasone, Cefotaxime, Imipenem, Ganciclovir	Improved	(13)
P20	M	4y6m	*P. jirovecii, M. tuberculosis*, CMV	PCR	Fever, cough, diarrhea, bilateral lungs patchy infiltrates, respiratory failure, NS	–	Cotrimoxazole, Clindamycin, Primaquine, Ganciclovir	Improved	(14)
P21	F	12y	*P. jirovecii, Aspergillus fumigatus*	PCR	Acute chest pain, repeated pneumothorax, leukemia	–	ITZ, AmB, TMP-SMX	Improved	(15)
P22	F	7m	*P. jirovecii*, RHV	PCR	Fever, upper and lower respiratory tract infection, SCID	–	TMP-SMX	Improved	(16)
P23	M	9m	*P. jirovecii*, RHV	PCR	Lower respiratory tract infection, infantile NS	–	TMP-SMX	Improved	(16)
P24	F	6m	*P. jirovecii, P. aeruginosa* RHV	PCR	Lower respiratory tract infection, SCID	–	TMP-SMX	Improved	(16)
P25	F	6m	*P. jirovecii, S. pneumoniae*, *H. influenzae*, RHV	PCR	Lower respiratory tract infection, asthma	–	TMP-SMX	Improved	(16)
P26	M	4m	*P. jirovecii, H. influenzae*, RHV	PCR	Lower respiratory tract infection, hyaline membrane disease, pulmonary fibrosis	–	TMP-SMX	Improved	(16)
P27	F	9m	*P. jirovecii, S. pneumoniae*, *H. influenzae, M. catharalis*, RHV	PCR	Upper respiratory tract infection, infectious sequelae, asthma	–	TMP-SMX	Improved	(16)
P28	F	14y	*P. jirovecii, A. fumigatus*	Smear mNGS	Fever, cough, diffuse ground glass changes in the bilateral lungs, SLE	–	VCZ, TMP-SMX, Caspofungin acetate	Death	(17)
P29	F	8m	*P. jirovecii, Legionella pneumophila*	Culture PCR	Severe acute respiratory distress syndrome, multiorgan failure, infantile spasm	–	Ceftriaxone, Azithromycin	Death	(18)
P30	F	1y	*P. jirovecii, P. aeruginosa*, *S. aureus*	PCR	Fever, Upper and lower respiratory tract infection, Pierre Robin Syndrome	–	TMP-SMX	Death	(16)
P31	F	3m	*P. jirovecii, H. influenzae*	PCR	Lower respiratory tract infection, right-sided pleural effusions, cardiopathy	–	TMP-SMX	Death	(16)

EBV, Epstein-Barr virus; RHV, Rhinovirus; Cytomegalovirus, CMV; HBV, Hepatitis B virus; HSV, Herpes Simplex Virus; PCR, polymerase chain reaction; mNGS, metagenome next-generation sequencing; CMC, chronic mucocutaneous candidiasis; ARDS, acute respiratory distress syndrome; MODS, multiple organ dysfunction syndrome; HLH, hemophagocytic lymphohistiocytosis; ITP, immune thrombocytopenic purpura; NS, nephrotic syndrome; SCID, Severe combined immune deficiency; SLE, Systemic lupus erythematosus; ITZ, itraconazole; TMP-SMX, Trimethoprim-Sulfamethoxazole; VCZ voriconazole; AmB, amphotericin B.

Polymicrobial infections are important features of immunocompromised hosts and affect prognosis. Early and accurate pathogen diagnosis is particularly crucial in these patients. As the methods listed in **Table 1**, smear, culture, PCR, and mNGS are commonly used for pathogen detection. *T. marneffei* is usually diagnosed by microscopy and cultivation based on its morphological and dimorphic characteristics (19). Our patient was diagnosed with *T. marneffei* infection because of positive BALF smear, culture, and mNGS. Since *P. jirovecii* is hard to be cultured, definitive diagnosis requires detection and identification of the organism mainly by dye staining or PCR (2, 17). In this case, the *P. jirovecii* infection was diagnosed by PCR and mNGS assays of BALF. *T. marneffei* and *P. jirovecii* were identified in one test of mNGS, but not accomplished in one assay of culture, smear, or PCR. Considering the high risk of mixed infection in immunocompromised individuals, timely use of mNGS could play a positive role in avoiding missed diagnoses and improving prognosis (20).

*T.marneffei* mainly causes upper or lower respiratory infection, especially pulmonary infection, in immunocompromised individuals with HIV infection or functional impairments of cellular immunity (21). The dimorphic ability of *T.marneffei* to switch from environmental mycelium to parasitic yeast form is recognized as a challenging virulence factor to host immune defenses (1). *P. jirovecii* most commonly affects the respiratory function of immunocompromised patients, possibly with nonspecific signs of fever, cough, and dyspnea (2). Adherence of *P. jirovecii* to alveoli and the host’s inflammatory response are the main reasons causing significant lung injury, hypoxia, or even respiratory failure (2). Except for the common symptoms of fever and pneumonia in fungi infection, our patient manifested trachyphonia. The inner tracheal membrane was the rare infection site for these two pathogens, thus, accumulating experience of the infection sites and manifestations is beneficial for promoting early diagnosis and timely therapy.

*T.marneffei* and *P. jirovecii* are opportunistic pathogenic fungi that have a major impact on immunocompromised patients. This boy was diagnosed with primary immunodeficiency caused by *STAT1* R274W mutation, with proofs of WES and sanger sequencing. Among the mutation regions in *STAT1*, the 274th amino acid of arginine (R274), which is in the coiled-coil domain, is one of the most common mutation sites found in more than 70 patients (4, 22–24). The *STAT* family members can be activated through phosphorylation. Briefly, they are phosphorylated by the receptor-associated kinases, then form homodimers or heterodimers that translocate from the cytoplasm to the nucleus and bind to the specific DNA consensus sequences to induce target gene transcription. Additionally, *STAT1* influences the transcription of *STAT3*-inducible genes, as *STAT1* and *STAT3* compete for the DNA-binding sites (25). *STAT1* R274W mutation leads to an increased phosphorylated *STAT1*, thus, called gain-of-function (GOF) mutation (26). In line with the abundant downstream genes regulated by the *STAT* family, the clinical spectrum associated with immunodeficient patients carrying *STAT1* mutation was unexpectedly broad (4, 27). In statistics of more than 250 *STAT1* GOF patients, most *STAT1* patients had normal total T (75.6%) and CD4+ T (68.1%) lymphocytes, only a few patients showed increased total T (1.4%) and CD4+ T (1.1%) lymphocytes (28). Leiding analyzed one *STAT1* R274W case, diagnosed with chronic mucocutaneous candidiasis, mycotic cerebral aneurysms, and pneumonia (caused by *H. influenzae*, *P. aeruginosa*, *S. pneumoniae*), showing T cell lymphopenia (24). Different from the observations of Leiding, our patient had normal T lymphocyte counts but increased CD4+ T cells. In a case review, 87.8% of the 90 patients with *STAT1* GOF mutation showed Th17 cytopenia, and the remaining 12.2% of patients presented normal levels of Th17 cells (28). Similar to most cases, the boy had decreased Th17 of CD3+. The GOF mutation can decrease IL-17 secretion through two mechanisms, 1) directly inhibits the differentiation of T cells into Th17 cells; 2) impairs the pathway that IL-6, IL-21, and IL-23 induce Th17 cell differentiation through *STAT3* (29). The decreased Th17 differentiation impairs IL-17 function in the defense against extracellular pathogens like fungi, which might explain the susceptibility of our patient to *T.marneffei* and *P. jirovecii* (29, 30). Interestingly, the CD4+ subset analysis was also performed in our patient, and the decreased CD4+ effector memory (EM) was observed, which might be following one of the differentiation models that CD4+ EM are generated from Th17 (31). However, the roles and biology of memory CD4+ cells are complex and less well understood. There are 32.1% of 209 *STAT1* GOF patients with a reduced percentage of NK cells and 1.4% with increased NK cells, while most cases showed normal NK cells (28). In this study, the declined NK cells were consistent with a few cases. The impaired NK cell proliferation was associated with increased *STAT1* phosphorylation and reduced *STAT5* activation in NK cells of *STAT1* GOF patients (32). NK lymphocytes confer a primary immune response against intracellular pathogens and virally infected cells. Therefore, our patient’s severely reduced NK cells indicated an impaired defense against intracellular *T.marneffei* (1, 32). In the 264 *STAT1* GOF patients summarized by Zhang, 74.2% had normal B lymphocytes (28). Consistently, our patient presented normal B lymphocytes. Among the 63 *STAT1* GOF patients for whom memory B cell data were available, 50.8% had a reduced memory B lymphocyte subset (28). Our patient presented lower memory B lymphocytes and impaired B-cell differentiation, common with a *STAT1* R274W patient with disseminated Cryptococcosis (22). Since the activation of *STAT1*, *STAT3*, and *STAT5* is fundamental for the differentiation of human B cells into memory cells, the B cell differentiation might be impaired by the higher level of *STAT1* phosphorylation in *STAT1* GOF patients (33, 34). Although reported *STAT1* cases are increasing, there have been no reports of *T.marneffei* and *P. jirovecii* coinfection. The immune responses of our *STAT1* GOF patient illustrated the complexity of *STAT1-* associated immunodeficiency, which needs additional research.

The treatment for mixed infection was challenging and lacked a standard. Amphotericin B is highly effective as induction therapy for *T.marneffei* infection, but can cause serious adverse effects, such as liver and kidney damage and severe hypokalemia (35). Voriconazole and itraconazole are more frequently used in children for anti-fungal therapy and have been confirmed to be safe and effective (36, 37). The first-line treatment choice for *P. jirovecii* pneumonia is trimethoprim-sulfamethoxazole (2). Considering the severely mixed fungi infection and the persistent fungal susceptibility in primary immunodeficient patients, the boy was given long-term itraconazole and trimethoprim-sulfamethoxazole as the dominating treatments for therapy and precaution (38). The subsequent anti-bacterial therapy was short-term due to the low copy numbers of *S. pneumoniae* and the anti-bacterial treatments administered before. The child improved significantly and showed no recurrent infections in the one-year follow-up, which suggested a successful therapy for unusual mixed fungi infection.

## Conclusion

When anti-infective treatment is ineffective, pathogens are hard to be detected by conventional methods. It is necessary to consider opportunistic pathogen infections. mNGS can rapidly and accurately identify the pathogen, especially for the mixed infections, helping clinical decision-making. When *T. marneffei* and *P. jirovecii* co-infection occurs, a genetic test should be taken to discover underlying immunodeficiency disease, achieve an early diagnosis, and improve the patient’s prognosis.

## Data availability statement

The original contributions presented in the study are included in the article/**Supplementary Material**. Further inquiries can be directed to the corresponding authors.

## Ethics statement

The studies involving human participants were reviewed and approved by Ethics Committee of Shenzhen Children’s Hospital. Written informed consent to participate in this study was provided by the participants’ legal guardian/next of kin.

## Author contributions

QY and CY analyzed data and wrote the paper. YW, KC, XL and WC collected patients’ clinical data and modified the paper. LC, YB and SZ made the figures and tables. WW, YZ and HZ supervised the whole writing process. All authors contributed to the article and approved the submitted version.

## Supplementary material

Supplementary Table 1 The results of flow cytometry for immune cells.
